# Early prediction of pulmonary outcomes in preterm infants using electrical impedance tomography

**DOI:** 10.3389/fped.2023.1167077

**Published:** 2023-05-24

**Authors:** Vincent D. Gaertner, Tobias Mühlbacher, Andreas D. Waldmann, Dirk Bassler, Christoph M. Rüegger

**Affiliations:** ^1^ Newborn Research Zurich, Department of Neonatology, University Hospital and University of Zurich, Zurich, Switzerland; ^2^ Department of Neonatology, Dr von Hauner University Children's Hospital, Ludwig-Maximilian-University, Munich, Germany; ^3^ Department of Anesthesiology and Intensive Care Medicine, Rostock University Medical Center, Rostock, Germany

**Keywords:** neonatology, bronchopulmonary dysplasia, electrical impedance tomograghy, preterm infants, surfactant nebulization, respiratory support, prediction

## Abstract

**Introduction:**

Electrical impedance tomography (EIT) allows assessment of ventilation and aeration homogeneity which may be associated with respiratory outcomes in preterm infants.

**Methods:**

This was a secondary analysis to a recent randomized controlled trial in very preterm infants in the delivery room (DR). The predictive value of various EIT parameters assessed 30 min after birth on important respiratory outcomes (early intubation <24 h after birth, oxygen dependency at 28 days after birth, and moderate/severe bronchopulmonary dysplasia; BPD) was assessed.

**Results:**

Thirty-two infants were analyzed. A lower percentage of aerated lung volume [OR (95% CI) = 0.8 (0.66–0.98), *p *= 0.027] as well as a higher aeration homogeneity ratio (i.e., more aeration in the non-gravity-dependent lung) predicted the need for supplemental oxygen at 28 days after birth [9.58 (5.16–17.78), *p *= 0.0028]. Both variables together had a similar predictive value to a model using known clinical contributors. There was no association with intubation or BPD, where numbers were small.

**Discussion:**

In very preterm infants, EIT markers of aeration at 30 min after birth accurately predicted the need for supplemental oxygen at 28 days after birth but not BPD. EIT-guided individualized optimization of respiratory support in the DR may be possible.

## Introduction

Bronchopulmonary dysplasia (BPD) is among the major morbidities of very preterm infants (1, 2), and it is associated with adverse long-term neurodevelopmental outcomes as well as recurrent hospitalizations which is a large burden to infants and parents (3–5). Accurate prediction of a developing BPD may be important to guide early therapeutic interventions.

Currently, there are various tools to predict BPD, all of which lack accuracy (6). Recently, lung ultrasound has gained attention as a potentially novel tool to predict short- (surfactant need, intubation) and long-term (oxygen dependency, BPD) respiratory outcomes as early as 24 or 72 h after birth (7–10). However, at this timepoint, lung ultrasound adds only little additional predictive value to known clinical parameters such as gestational age (11). Also, lung ultrasound can only detect intrapulmonary consolidations and fluids close to the chest-wall and does not allow to draw conclusions on regional lung aeration.

Electrical impedance tomography (EIT) is a novel, noninvasive tool measuring regional ventilation distribution and overall lung aeration in a cross-sectional slice of the lung (12). Recently, ventilation inhomogeneity in stable preterm infants approximately four weeks after birth was associated with worse oxygenation and a later diagnosis of BPD (13). Using EIT, we aimed to describe whether parameters of inhomogeneous aeration and ventilation measured as early as 30 min after birth may already predict respiratory outcomes in very preterm infants and may add to currently known clinical predictive parameters.

## Methods

This is a secondary analysis of a prospective, parallel, randomized controlled trial conducted at the University Hospital Zurich comparing prophylactic surfactant nebulization (SN) immediately after birth to positive distending pressure alone (14). The original trial was registered with clinicaltrials.gov (NCT04315636) and approved by the local ethics committee (KEK-2020-00890). All parents provided antenatal written informed consent.

### Population and intervention

The setup of the original study has been described previously (14). In short, infants between 26 and 32 completed weeks of gestation at birth were randomized to either positive distending pressure alone after birth or positive distending pressure with additional SN (200 mg/kg Curosurf®, Chiesi Farmaceutici, Parma, Italy) via the eFlow® Neo Nebulizer (PARI Pharma, Starnberg, Germany) starting simultaneously with the initial application of a face mask. Infants were initially supported on continuous positive airway pressure support (CPAP) with a positive distending pressure of 8 mbar using the EVE NEO® ventilator (Fritz Stephan GmbH, Gackenbach, Germany) and a face mask (ComfortStar®, Dräger Medical System, Lübeck, Germany). Escalation of pressure levels and change of respiratory support mode or interface were possible at any time during stabilization at the clinician's discretion. To allow comparability, infants who had received intratracheal surfactant (via endotracheal tube or via thin catheter) within the first 30 min after birth were excluded.

### Data collection

Complete methods of data collection have been described previously (14). A researcher was present for each delivery to fasten a textile EIT belt at nipple level as soon as the infant reached the resuscitaire. The LuMon^™^ device (SenTec AG, Landquart, Switzerland) was used to record EIT data at a frame rate of 51 Hz (15, 16). The EIT belt remained on the infant's thorax for the initial 90 min or until the first chest x-ray was performed. Thirty minutes after birth, data were extracted for 30 s of artefact-free tidal ventilation using ibeX (version 1.1, SenTec AG, Landquart, Switzerland). Infants were lying in supine position during all measurements. Thus, dorsal lung regions were considered gravity-dependent (17, 18).

### Predictors

We aimed to show the early predictive value of EIT parameters on short- and long-term pulmonary outcomes. Therefore, we chose to evaluate data at 30 min after birth as infants are largely transitioned at this timepoint while it still allows early prediction.

EIT data were analyzed using Matlab software (version 2019a, Mathworks, Nantick, MA, USA). Recently, heterogeneous ventilation in stable preterm infants was associated with a later diagnosis of BPD (13). Building on this finding, we pre-defined the following parameters which may influence pulmonary outcomes: The percentage of overall aerated lung volume (Aer%; as indicator of overall aeration), the aeration homogeneity ratio (AHR; as indicator of aeration homogeneity), the coefficient of variation (CV; as indicator of ventilation homogeneity), as well as the gravity- and non-gravity-dependent silent spaces (SS_GD_ and SS_NGD_; as indicators of atelectasis and overdistension, respectively).

To obtain these predictors, the following steps of analysis were necessary: first, data was prepared by projecting predefined anatomical lung regions based on the vendor-provided human model chest atlas into the EIT image, excluding EIT signals outside of these regions and normalizing the signal for body weight (17–19). Second, the net EIT signal at end-expiration (end-expiratory lung impedance; EELI) was isolated in arbitrary units (AU) in the entire lung and the percentage of aerated lung tissue was calculated (Aer%). Then, aeration in the non-gravity-dependent and the gravity-dependent lung (EELI_NGD_, EELI_GD_) was isolated and the aeration homogeneity ratio (AHR) was calculated by dividing the weighted values of the non-gravity-dependent half of the EIT signal by the gravity-dependent half (18, 20). A higher value indicates more aeration in the non-gravity-dependent lung (18). Third, the CV was calculated by dividing the standard deviation (SD) of impedance changes in all pixels by the mean value of impedance (18, 21). The CV correlates with ventilation homogeneity and lower values indicate improved homogeneity. Finally, silent spaces were calculated in the gravity- and non-gravity-dependent lung [corresponding to atelectasis (SS_GD_) and overdistension (SS_NGD_)] (12, 18, 22). As percentages, SS were not normalized for body weight.

### Outcomes and timepoints

The following outcomes were assessed: First, early respiratory failure, defined as either endotracheal intubation (with subsequent surfactant application) or less invasive surfactant application (LISA) within the first 24 h after birth, as both manipulations would severely change ventilation and aeration parameters. Second, oxygen dependency at 28 days, irrespective of the mode of respiratory support. And third, bronchopulmonary dysplasia (BPD), defined as oxygen need or any pressure support at 36 weeks postmenstrual age.

### Statistical analysis

Averages of each EIT recording were computed for subsequent analyses. Normally distributed data are presented as mean with standard deviation (SD) or 95% confidence interval (CI). Non-parametric data are presented as median and interquartile range (IQR).

Logistic regression was performed for each predictor to assess the effect of EIT variables on respiratory outcomes. The area under the receiver operating curve (ROC) was then calculated for significant predictors of respiratory outcomes using the pROC package in R statistics. Finally, significant predictors were included into a model predicting adverse outcomes using three known clinical contributors (gestational age, sex and birth weight) to assess whether there is an improvement in the prediction. *P*-values < 0.05 were considered statistically significant. All analyses were performed using R statistics (version 3.6.2) (23).

## Results

### Population

Overall, 35 infants were randomized in the original study, two of which were intubated before 30 min and 1 had a faulty EIT recording, leaving 32 infants for data analysis. Demographic characteristics can be found in Table 1.

**Table 1:** Demographic characteristics of the study population.

Patient characteristics	Included patients (*N* = 32)
Demographic	
Gestational age at birth (weeks)	29.6 (28.7–31.1)
Birth weight (g)	1140 (889–1368)
Male, *n* (%)	16 (50%)
Prenatal	
Completed antenatal steroids, *n* (%)	23 (72%)
PPROM, *n* (%)	9 (28%)
Chorioamnionitis, *n* (%)	5 (16%)
Oligo- or anhydramnios, *n* (%)	6 (19%)
Preeclampsia, *n* (%)	10 (31%)
IUGR, *n* (%)	8 (25%)
Delivered by CS, *n* (%)	30 (94%)
Postnatal	
Time of cord clamping (s)	60 (49–60)
Apgar score at 5 min	8 (7–9)
Umbilical artery pH	7.32 (7.28–7.35)
Doses of surfactant	1 (0–1)
Sepsis, *n* (%)	2 (6%)
Respiratory outcomes	
Respiratory failure (intubation/LISA)	
In the delivery room, *n* (%)	1 (1/0; 3%)
Within 24 h, *n* (%)	7 (2/5; 22%)
Within 72 h, *n* (%)	11 (6/5; 34%)
During hospitalization, *n* (%)	11 (6/5; 34%)
Bronchopulmonary dysplasia (Walsh) (24)	
Any, *n* (%)	15 (47%)
Mild, *n* (%)	14 (44%)
Moderate or severe, *n* (%)	1 (3%)

Median (IQR) are shown except where otherwise specified. PPROM, prolonged premature rupture of the membranes; IUGR, intrauterine growth retardation; CS, cesarean section.

### Prediction of respiratory parameters by EIT variables

**Figure 1:**
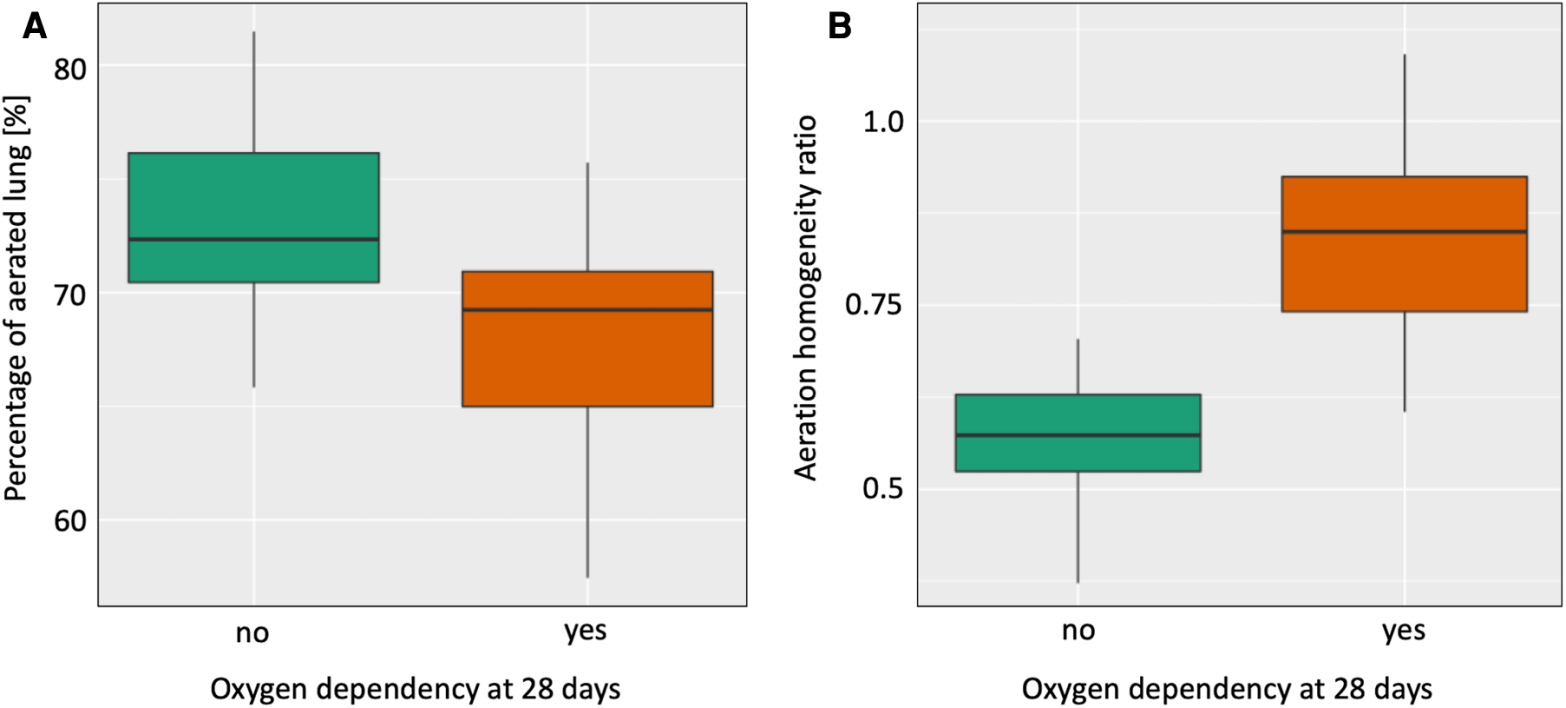
Differences in the percentage of overall lung aeration (Aer%, **A**) and the aeration homogeneity ratio (AHR, **B**) between infants who did vs did not receive supplemental oxygen at 28 days after birth.

None of the pre-specified EIT parameters predicted early intubation within the first 24 h after birth or moderate/severe bronchopulmonary dysplasia (Table 2). A lower percentage of overall aerated lung [OR (95% CI) = 0.8 (0.66–0.98), ROC = 0.761, *p *= 0.027, Figure 1A] as well as a higher aeration homogeneity ratio (AHR) and thus, more aeration in the non-gravity-dependent lung predicted the need for supplemental oxygen at 28 days after birth [OR (95% CI) = 9.58 (5.16–17.78), ROC = 0.898, *p *= 0.0028, Figure 1B].

**Table 2:** Prediction of respiratory outcomes by pre-specified EIT parameters.

	Early intubation		O_2_ dependency at 28 days		Moderate/severe BPD	
	OR (95% CI)	*p*	OR (95% CI)	*p*	OR (95% CI)	*p*
Aer%	0.86 (0.72–1.03)	0.1252	**0.8** (**0.66**–**0.98)**	**0**.**0272**	1.13 (0.79–1.6)	0.4856
AHR	0.85 (0.01–68.74)	0.9424	**9.58** (**5.16**–**17.78)**	**0**.**0028**	11.0 (0.01–17.29)	0.3407
CV	2.92 (0.08–109.51)	0.5636	1.57 (0.07–34.7)	0.7756	31.2 (0.01–1084.0)	0.4084
SS_NGD_	1.02 (0.79–1.32)	0.8483	0.97 (0.78–1.2)	0.7549	1.02 (0.57–1.84)	0.9566
SS_GD_	0.76 (0.43–1.33)	0.3257	1.23 (0.83–1.83)	0.3013	3.13 (0.48–20.52)	0.2383

Bold values indicate significant differences.

CV, coefficient of variation; SS_GD_, gravity-dependent silent spaces; AHR, aeration homogeneity ratio; SS_NGD_, non-gravity-dependent silent spaces; Aer%, percentage of overall aerated lung tissue; OR, odds ratio; CI, confidence interval.

### Prediction models including clinical parameters

**Figure 2:**
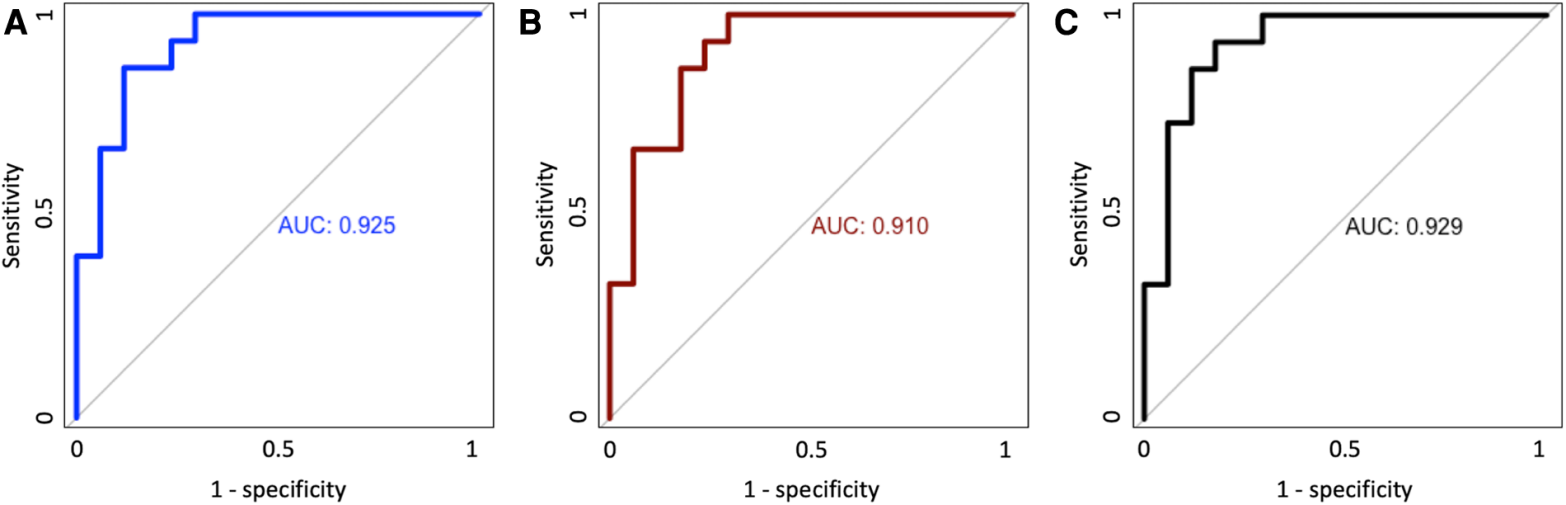
Area under the receiver operating curve (ROC) for clinical variables alone (**A**), EIT variable alone (**B**) and the combination of clinical and EIT variables (**C**).

Predicting oxygen need at 28 days after birth, the two EIT variables combined had a similar predictive value [area under the receiver operating curve (ROC) = 0.925] as a model including known contributing clinical parameters (sex, weight and gestational age: ROC = 0.910). Adding the two EIT variables to the model of clinical parameters alone improved the ROC only slightly (ROC = 0.929). All models provided a near perfect prediction of oxygen dependency at 28 days (Figures 2A–C).

## Discussion

In a small sample of very preterm infants, EIT markers of aeration and ventilation at 30 min after birth could not predict early intubation or bronchopulmonary dysplasia. However, EIT markers of aeration, i.e., a lower percentage of lung aeration as well as a higher aeration homogeneity ratio, measured 30 min after birth predicted the need for supplemental oxygen at 28 days after birth. The predictive value of EIT parameters was similar to a model including clinical parameters.

Electrical impedance tomography is a relatively novel tool which provides functional images of ventilation and aeration (12), and is representative for the entire lung in ventilated preterm infants (24). Recently, heterogeneous ventilation, measured by EIT, was associated with poor oxygenation and a later diagnosis of BPD in stable preterm infants approximately four weeks after birth (13). In this small pilot analysis, we did not see an association of EIT parameters with early intubation and subsequent surfactant application. However, we saw that the percentage of aerated lung as well as a higher aeration homogeneity ratio (AHR) predicted the need for oxygen at 28 days after birth. Early intubation is mostly performed in case of poor respiratory drive or insufficient oxygenation which may be compensated initially, e.g., by the use of noninvasive positive pressure ventilation and/or increased pressure levels. Infants who were not intubated within the first 24 h could still go on to require oxygen at 28 days after birth due to inflammatory processes which may explain why we only saw an association of EIT parameters with oxygen dependency.

It is conceivable that infants with an improved overall aeration at 30 min, indicated by a large percentage of aerated lung, may require less respiratory support. This in turn is associated with an improved respiratory outcome (25), and may explain the association with oxygen dependency at 28 days. At the same time, a higher AHR, which corresponds to more aeration in the non-gravity-dependent part of the lung when compared with the gravity-dependent part, was associated with a higher likelihood of requiring oxygen 28 days after birth. Thus, overdistension of the lung (indicated by an increase in AHR) may be injurious to the lung which may explain the inverse association of the AHR with oxygen dependency. Finding the optimal balance between too little aeration and overdistending the lung remains challenging. Targeting the ideal PEEP level is of utmost importance to guide early respiratory interventions during the transitional period of extremely preterm infants. Accordingly, currently ongoing trials are targeting an individualized approach to pressure support immediately after birth (26). We speculate that EIT may be a helpful tool to guide respiratory support in the delivery room and beyond but future prospective studies need to evaluate this before a recommendation can be made.

Interestingly, these two EIT variables predicted oxygen dependency at 28 days after birth similarly good as clinical parameters (27). While EIT added little predictive value to clinical parameters, our finding underlines the importance of establishing functional residual capacity after birth not only for the immediate adaptation after birth but also for medium-term respiratory outcomes. However, our data is based on a limited number of infants in a single centre and results may differ in other settings.

While oxygen dependency in very preterm infants is largely explained by gestational age and birth weight, BPD is influenced by more factors than basic demographic characteristics and thus, early prediction of BPD by clinical parameters is still inaccurate (6). However, a late prediction is not as useful because therapeutic options are more effective in the first days after birth (28). While we saw an association of EIT parameters on medium-term respiratory outcome, we did not find an association with the development of BPD in this small study. Thus, it remains unclear whether EIT can add to the existing tools to predict BPD. While there was no clear association, the effect direction was similar to the effects found on oxygen dependency at 28 days after birth. Factors associated with mild BPD may also be associated with moderate or severe BPD in a larger cohort. It is important to note that we only had one patient who developed moderate or severe BPD and subsequent lack of power precluded meaningful analyses. Recently, lung ultrasound has gained attention as a potentially novel tool to predict BPD as early as 24 or 72 h after birth (7–10). However, at this timepoint, there are various clinical parameters allowing a fairly accurate prediction of BPD and lung ultrasound adds only little additional predictive value (11). While lung ultrasound only detects intrapulmonary consolidations and fluids, EIT could add to this knowledge by describing lung aeration (12). We speculate that the combination of lung ultrasound and EIT may allow a good prediction of BPD even as early as 30 min after birth but prospective clinical studies with a larger sample size are needed to evaluate this hypothesis. As BPD is a multifactorial disease, an all-encompassing approach may be needed which includes clinical and visualization (EIT, lung ultrasound) parameters as well as biomarkers (29) and machine-learning (30) to accurately predict this important outcome for patients and their families.

This study has limitations: First, it was a single-center study and it is unclear whether different approaches to airway management in other centres may have yielded different results. Second, it is a secondary analysis of a prospective trial on the effect of surfactant nebulization which is not standard of care and may have skewed the data. Third, it was a small trial with only 32 infants included in the analysis. This precluded meaningful analysis of the most important respiratory outcome in the NICU, BPD. However, this is the largest prospective trial with EIT measurements in preterm infants immediately after birth and it provides a first idea of the predictive value of EIT parameters after birth. Larger prospective trials, possibly including lung ultrasound as well as EIT, are needed to accurately describe their additional predictive value.

## Conclusion

In very preterm infants, EIT markers of aeration and ventilation at 30 min after birth could not predict early intubation or bronchopulmonary dysplasia. However, EIT markers of aeration, i.e., a lower percentage of lung aeration as well as a higher aeration homogeneity ratio at 30 min after birth, accurately predicted the need for supplemental oxygen at 28 days after birth. In fact, these two EIT parameters had a similar predictive value to a model including known contributing clinical parameters. Our results highlight the importance of an individualized approach to respiratory support in very preterm infants after birth, and EIT may be useful to guide respiratory support in this situation.

## Data Availability

Deidentified individual participant data will be made available from three months to three years following publication to researchers who provide a methodologically sound proposal, with approval by an independent review committee (“learned intermediary”). Data requestors will need to sign a data access or material transfer agreement approved by USZ. Proposals should be submitted to VG, vincent.gaertner@usz.ch.

## References

[1] Jensen EA Edwards EM Greenberg LT Soll RF Ehret DEY Horbar JD. Severity of bronchopulmonary dysplasia among very preterm infants in the United States. Pediatrics. (2021) 148(1):e2020030007. 10.1542/peds.2020-030007 34078747 PMC8290972

[2] Avila-Alvarez A Zozaya C Pértega-Diaz S Sanchez-Luna M Iriondo-Sanz M Elorza MD Temporal trends in respiratory care and bronchopulmonary dysplasia in very preterm infants over a 10-year period in Spain. Arch Dis Child Fetal Neonatal Ed. (2022) 107:143–9. 10.1136/archdischild-2021-322402 34321246

[3] Martin M Smith L Hofheimer JA McGowan EC O'Shea TM Pastyrnak S Bronchopulmonary dysplasia and neurobehavioural outcomes at birth and 2 years in infants born before 30 weeks. Arch Dis Child Fetal Neonatal Ed. (2022) 108(2):142–8. 10.1136/archdischild-2021-323405 35999044 PMC9947192

[4] Cheong JLY Doyle LW. An update on pulmonary and neurodevelopmental outcomes of bronchopulmonary dysplasia. Semin Perinatol. (2018) 42:478–84. 10.1053/j.semperi.2018.09.013 30401478

[5] Sillers L Alexiou S Jensen EA. Lifelong pulmonary sequelae of bronchopulmonary dysplasia. Curr Opin Pediatr. (2020) 32:252–60. 10.1097/MOP.0000000000000884 32084032

[6] Onland W Debray TP Laughon MM Miedema M Cools F Askie LM Clinical prediction models for bronchopulmonary dysplasia: a systematic review and external validation study. BMC Pediatr. (2013) 13:207. 10.1186/1471-2431-13-207 24345305 PMC3878731

[7] Pezza L Alonso-Ojembarrena A Elsayed Y Yousef N Vedovelli L Raimondi F Meta-analysis of lung ultrasound scores for early prediction of bronchopulmonary dysplasia. Ann Am Thorac Soc. (2022) 19:659–67. 10.1513/AnnalsATS.202107-822OC 34788582

[8] Alonso-Ojembarrena A Serna-Guerediaga I Aldecoa-Bilbao V Gregorio-Hernández R Alonso-Quintela P Concheiro-Guisán A The predictive value of lung ultrasound scores in developing bronchopulmonary dysplasia: a prospective multicenter diagnostic accuracy study. Chest. (2021) 160:1006–16. 10.1016/j.chest.2021.02.066 33689782

[9] Loi B Vigo G Baraldi E Raimondi F Carnielli VP Mosca F Lung ultrasound to monitor extremely preterm infants and predict bronchopulmonary dysplasia. A multicenter longitudinal cohort study. Am J Respir Crit Care Med. (2021) 203:1398–409. 10.1164/rccm.202008-3131OC 33352083

[10] Mohamed A Mohsen N Diambomba Y Lashin A Louis D Elsayed Y Lung ultrasound for prediction of bronchopulmonary dysplasia in extreme preterm neonates: a prospective diagnostic cohort study. J Pediatr. (2021) 238, 187–92.e2. 10.1016/j.jpeds.2021.06.079 34237347

[11] Woods PL Stoecklin B Woods A Gill AW. Early lung ultrasound affords little to the prediction of bronchopulmonary dysplasia. Arch Dis Child Fetal Neonatal Ed. (2021) 106:657–62. 10.1136/archdischild-2020-320830 33990385

[12] Frerichs I Amato MBP van Kaam AH Tingay DG Zhao Z Grychtol B Chest electrical impedance tomography examination, data analysis, terminology, clinical use and recommendations: consensus statement of the TRanslational EIT developmeNt stuDy group. Thorax. (2017) 72:83–93. 10.1136/thoraxjnl-2016-208357 27596161 PMC5329047

[13] Thomson J Rüegger CM Perkins EJ Pereira-Fantini PM Farrell O Owen LS Regional ventilation characteristics during non-invasive respiratory support in preterm infants. Arch Dis Child Fetal Neonatal Ed. (2021) 106:370–5. 10.1136/archdischild-2020-320449 33246967

[14] Gaertner VD Minocchieri S Waldmann AD Mühlbacher T Bassler D Rüegger CM Prophylactic surfactant nebulisation for the early aeration of the preterm lung: a randomised clinical trial. Arch Dis Child Fetal Neonatal Ed. (2022) 108(3):217–23. 10.1136/archdischild-2022-324519 36424125

[15] Plastina L Gaertner VD Waldmann AD Thomann J Bassler D Rüegger CM. The DELUX study: development of lung volumes during extubation of preterm infants. Pediatr Res. (2021) 92(1):242–48. 10.1038/s41390-021-01699-w 34465873 PMC8406659

[16] Gaertner VD Waldmann AD Bassler D Hooper SB Rüegger CM. Intrapulmonary volume changes during hiccups versus spontaneous breaths in a preterm infant. Neonatology. (2022) 119(4):1–5. 10.1159/000524194 35398844

[17] Gaertner VD Waldmann AD Davis PG Bassler D Springer L Thomson J Transmission of oscillatory volumes into the preterm lung during noninvasive high-frequency ventilation. Am J Respir Crit Care Med. (2021) 203:998–1005. 10.1164/rccm.202007-2701OC 33095994

[18] Gaertner VD Waldmann AD Davis PG Bassler D Springer L Thomson J Lung volume distribution in preterm infants on non-invasive high-frequency ventilation. Arch Dis Child Fetal Neonatal Ed. (2022) 107(5):551–57. 10.1136/archdischild-2021-322990 35101993

[19] Tingay DG Waldmann AD Frerichs I Ranganathan S Adler A. Electrical impedance tomography can identify ventilation and perfusion defects: a neonatal case. Am J Respir Crit Care Med. (2019) 199:384–6. 10.1164/rccm.201808-1551LE 30365365

[20] Tingay D Rajapaksa A Zonneveld C Black D Perkins E Adler A Spatiotemporal aeration and lung injury patterns are influenced by the first inflation strategy at birth. Am J Respir Cell Mol Biol. (2016) 54:263–72. 10.1165/rcmb.2015-0127OC 26186685

[21] Becher T Vogt B Kott M Schädler D Weiler N Frerichs I. Functional regions of interest in electrical impedance tomography: a secondary analysis of two clinical studies. PLoS One. (2016) 11:e0152267. 10.1371/journal.pone.0152267 27010320 PMC4806869

[22] Spadaro S Mauri T Böhm SH Scaramuzzo G Turrini C Waldmann AD Variation of poorly ventilated lung units (silent spaces) measured by electrical impedance tomography to dynamically assess recruitment. Crit Care. (2018) 22(1):26. 10.1186/s13054-017-1931-7 29386048 PMC5793388

[23] R Core Team. R: A language and environment for statistical computing. Vienna, Austria: R Foundation for Statistical Computing. (2013).

[24] van der Burg PS Miedema M de Jongh FH Frerichs I van Kaam AH. Cross-sectional changes in lung volume measured by electrical impedance tomography are representative for the whole lung in ventilated preterm infants. Crit Care Med. (2014) 42:1524–30. 10.1097/CCM.0000000000000230 24561568

[25] Schmölzer GM Kumar M Pichler G Aziz K O'Reilly M Cheung PY. Non-invasive versus invasive respiratory support in preterm infants at birth: systematic review and meta-analysis. Br Med J. (2013) 347:f5980. 10.1136/bmj.f5980 24136633 PMC3805496

[26] clinicaltrials.gov. Positive end-expiratory pressure (PEEP) levels during resuscitation of preterm infants at birth (The POLAR Trial). (2022) [cited 2022 Nov 26]. Available from: https://clinicaltrials.gov/ct2/show/NCT04372953

[27] Hentschel J Friedel C Maier RF Bassir C Obladen M. Predicting chronic lung disease in very low birthweight infants: comparison of 3 scores. J Perinat Med. (1998) 26:378–83. 10.1515/jpme.1998.26.5.378 10027133

[28] Gilfillan M Bhandari A Bhandari V. Diagnosis and management of bronchopulmonary dysplasia. Br Med J. (2021) 375:n1974. 10.1136/bmj.n1974 34670756

[29] Lal CV Biomarkers AN Diagnosis E. And clinical predictors of bronchopulmonary dysplasia. Clin Perinatol. (2015) 42:739–54. 10.1016/j.clp.2015.08.004 26593076 PMC4662054

[30] Leigh RM Pham A Rao SS Vora FM Hou G Kent C Machine learning for prediction of bronchopulmonary dysplasia-free survival among very preterm infants. BMC Pediatr. (2022) 22:542. 10.1186/s12887-022-03602-w 36100848 PMC9469562

